# The effect of pre‐exercise hyperventilation on repeated high‐intensity inclined sprint performance

**DOI:** 10.1113/EP093393

**Published:** 2026-05-25

**Authors:** Leon Fesseler, Andreas Patzak, Viktor Heinz, Ngan‐Ha Stella Le, Oliver Opatz, Niklas Pilz, Tomas Lucca Bothe

**Affiliations:** ^1^ Institute of Physiology Center for Space Medicine and Extreme Environments Berlin Charité – Universitätsmedizin Berlin, corporate member of Freie Universität Berlin and Humboldt‐Universität zu Berlin Berlin Germany; ^2^ Institute of Translational Physiology Charité – Universitätsmedizin Berlin, corporate member of Freie Universität Berlin and Humboldt‐Universität zu Berlin Berlin Germany; ^3^ Institute of Integrative NeuroAnatomy Center for Space Medicine and Extreme Environments Germany Charité – Universitätsmedizin Berlin, corporate member of Freie Universität Berlin and Humboldt‐Universität zu Berlin Berlin Germany; ^4^ Department of Cardiology and Angiology Hannover Medical School Hannover Germany; ^5^ Institute of Sport Science Leibnitz University Hannover Hannover Germany; ^6^ Menzies Institute for Medical Research University of Tasmania Hobart Australia; ^7^ Berlin Fire Brigade Staff Division Research Projects Berlin Germany

**Keywords:** hyperventilation, respiratory alkalosis, sprint interval training

## Abstract

High‐intensity exercise induces systemic acidosis, which contributes to muscular performance decline. Controlled pre‐exercise hyperventilation has been proposed as a non‐invasive intervention to induce respiratory alkalosis and counteract acidosis‐related acute fatigue. In this study, we hypothesized that a real‐world‐applicable pre‐exercise hyperventilation protocol can improve incline sprint performance. This randomized, single‐centre, controlled crossover study included 36 recreational athletes aged 18–40 years. Each participant completed a series of three inclined treadmill interval sprints on two separate days: one with pre‐exercise hyperventilation and one without. Respiratory parameters were measured via spirometry, and post‐sprint capillary blood gas analysis was performed. Pre‐exercise hyperventilation resulted in higher mean CO_2_ elimination during hyperventilation (32.8 ± 5.1 vs. 21.5 ± 2.5 mL/kg, *P* < 0.0001) and lower mean end‐tidal CO_2_ partial pressure (17 ± 2.5 mmHg vs. 30 ± 2.7 mmHg, *P* < 0.0001) compared to control condition. Mean cumulative elevation gain did not differ between pre‐exercise hyperventilation (83.2 ± 27.6 m) and control condition (84.3 ± 26.0 m, *P* = 0.6461). Differences in CO_2_ elimination did not correlate with differences in performance (*P* = 0.5905). We found no alterations in post‐sprint capillary blood lactate and pH between the two conditions (*P* > 0.05 for all sprints cumulatively and separately). Participants subjectively assessed the breathing protocol as unpleasant, and felt it negatively influenced their performance. The easily translatable pre‐exercise hyperventilation protocol investigated in this study did not improve performance in repeated high‐intensity incline sprint intervals. Therefore, we discourage its application in real‐world applications.

## Abstract (highlights)

- **What is the central question of this study?**
  Does a real‐world‐applicable pre‐exercise hyperventilation protocol improve incline sprint performance?
- **What is the main finding and its importance?**
  A pre‐exercise hyperventilation protocol designed for real‐world implementation did not improve performance during repeated high‐intensity inclined treadmill sprints in young adult athletes. In addition, no alterations were found in post‐sprint capillary blood lactate and pH when comparing pre‐exercise hyperventilation with the control condition. Therefore, these findings do not support the use of this pre‐exercise hyperventilation protocol for performance enhancement in comparable athletic settings.

## 1 INTRODUCTION

Both sprinting and high‐intensity interval training (HIIT) induce a systemic acidic shift in working skeletal muscles. Metabolic acidosis inhibits phosphofructokinase, thereby impairing glycolytic flux (Cairns & Lindinger, 2025; Zagatto et al., 2022). Additionally, acidosis reduces calcium sensitivity of myofibrils and disrupts calcium release from the sarcoplasmic reticulum, perturbing excitation–contraction coupling (Unger & Debold, 2019; Zagatto et al., 2022). Taken together, these effects constitute a major limiting factor for muscle performance during intense exercise (Allen et al., 2008; Hollidge‐Horvat et al., 1999). Beyond the myocyte, extracellular acidosis can further constrain performance by altering ion channel behaviour and neuromuscular transmission (Blaustein et al., 2020). It further activates metabolite‐sensitive group III/IV afferents that heighten perceived effort and may restrict central motor drive in extreme environments (Cairns & Lindinger, 2025; Deb et al., 2018). Within the circulatory system, it modifies blood buffering and O_2_ transport dynamics via the Bohr effect (Mairbäurl, 2013). The ergogenic benefits of sodium bicarbonate supplementation in short‐duration, high‐intensity tasks underscore the functional relevance of these extracellular constraints (Grgic et al., 2021; Gurton et al., 2024) (Supporting Information).[Link], [Link]

With exercise bouts lasting between 30 s and 4 min, HIIT is a well‐established training modality that enhances both aerobic and anaerobic capacities as well as overall cardiovascular fitness (Engel et al., 2018; Milanović et al., 2015; Wu et al., 2021). At the same time, it mimics many real‐world use cases in which short‐term high performance intervals are of great operative importance such as for emergency responders (e.g., firefighters) or professional athletes (e.g., American football) (Stevenson et al., 2024; Williams‐Bell et al., 2010; Yue et al., 2025).

Controlled pre‐exercise hyperventilation has been suggested to increase blood pH by reducing CO_2_ levels (Dobashi et al., 2021; Sakamoto et al., 2014). It may delay the onset of exercise‐induced acidosis and attenuate performance decline during high‐intensity efforts (Leithäuser et al., 2016; Sakamoto et al., 2014). However, evidence regarding the efficacy of pre‐exercise hyperventilation remains inconsistent. Previous studies exploring this approach have been limited by small sample sizes and heterogeneous exercise protocols (Johnson et al., 2021; Leithäuser et al., 2016; Morrow et al., 1988; Sakamoto et al., 2014). Positive performance effects have primarily been reported in cycling‐based protocols employing repeated short‐duration sprints, such as 10 × 10 s efforts (Sakamoto et al., 2014). In contrast, several studies using comparable breathing protocols failed to demonstrate improvements in peak or mean power output, fatigue indices, or repeated sprint performance, particularly when different recovery structures, exercise durations, or exercise modalities were employed (Fujii et al., 2015; Leithäuser et al., 2016; Morrow et al., 1988)

Beyond its use in athletic contexts, controlled pre‐exercise hyperventilation may enhance performance in high‐demand occupational settings, such as rapid ascents during firefighting operations (Stevenson et al., 2024; Williams‐Bell et al., 2010). For practical implementation, the breathing protocol must be seamlessly integrated into existing workflows and demonstrate consistent reliability under real‐world conditions.

We hypothesized that an intensive bout of short duration hyperventilation can improve exercise performance during repeated incline sprints. The study design was chosen to provide directly translatable insights into real‐world performance needs as commonly experienced by emergency responders or athletes.

## 2 METHODS

### 2.1 Ethics

Ethical approval was obtained from the Ethics Committee Campus Charité Mitte of Charité—Universitätsmedizin Berlin (Approval No. EA1/254/23), and the trial was prospectively registered in the institution's clinical trial registry (ePA: 3000917) prior to initiating data collection. All procedures were carried out in accordance with the *Declaration of Helsinki* and German federal and EU legislation. Written informed consent was obtained from all participants before enrolment.

This study was conducted as a randomized, single‐centre, controlled crossover trial. We included recreational adult athletes aged up to 40 years who trained at least three times per week. The subjects had no relevant pre‐existing medical conditions and were not on regular medication (Table 1).

**TABLE 1:** Composition of the dataset (means ± SD).

	All (*n* = 36)	Male (*n* = 18)	Female (*n* = 18)
Age (years)	24.0 ± 1.8	24.7 ± 1.4	23.3 ± 1.9
Height (cm)	175.0 ± 9.7	182.6 ± 5.8	167.4 ± 6.2
Weight (kg)	69.6 ± 11.8	77.6 ± 10.6	61.5 ± 6.3
BMI (kg/m^2^)	22.6 ± 2.5	23.3 ± 2.8	21.9 ± 2.0
${\dot{V}}_{O_{2}\max}$ (ml/kg/min)	49.4 ± 8.0	54.2 ± 7.1	44.7 ± 5.7

Abbreviations: BMI, body mass index; ${\dot{V}}_{O_{2}\max}$, maximal oxygen uptake during 30 s rolling average.

### 2.2 Devices

#### 2.2.1 Anthropometry

Anthropometric data were obtained using the InBody 770 scale (InBody Co Ltd, Seoul, South Korea). After entering age, sex and height, the device measures impedance at six frequencies (1–1000 kHz) within approximately 60 s (Brewer et al., 2021).

#### 2.2.2 Treadmill

We used the Quasar Med 4.0 (H/P/Cosmos Sports & Medical GmbH, Nussdorf‐Traunstein, Germany) treadmill. The device allows a maximum speed of 25 km/h and a maximum grade of 28% including seven acceleration intensities. To minimize risk, participants were secured to the treadmill automatic switch‐off mechanism through a safety harness during all exercise bouts.

#### 2.2.3 Blood gas analysis

We performed capillary blood gas analysis (BGA) using the RAPIDPoint 500 (Siemens Healthineers, Erlangen, Germany). Quality control including device re‐calibrations were conducted three times daily (Nicolas et al., 2013).

#### 2.2.4 Spirometry

For continuous breath‐by‐breath monitoring of breathing gases, we used the METALYZER 3B (CORTEX Biophysik GmbH, Leipzig, Germany) spirometry with its accompanying MetaSoft Studio software (Version 5.16.0 SR2). The volume transducer as well as the gas analyser underwent daily calibrations according to the manufacturer's recommendations (Van Hooren et al., 2024).

### 2.3 Procedure

Each participant completed two experimental sessions in a randomized crossover design with a minimum break of 48 h in between, one with pre‐exercise hyperventilation and one without (control).

Anthropometric measurements were performed only in session 1. After attaching the spirometry mask and the safety harness to the participant, their left earlobe was treated with ‘Finalgon Wärmesalbe DUO’ (Finalgon, Frankfurt am Main, Germany), a rubefacient, to increase blood circulation and facilitate BGA. Following baseline assessments of capillary BGA and spirometry, a standardized 3‐min warm‐up was performed at 4 km/h and 10% inclined treadmill slope. Subsequently, we conducted another BGA at the beginning of the following 3‐min resting period. In the pre‐exercise hyperventilation condition, the last 30 s of rest included controlled hyperventilation.

The sprint protocol consisted of three incline sprints with strictly seated 3‐min recovery periods in between. We devised a modified Bruce protocol, which addresses potential real‐world application scenarios and ensures a comparable duration of exertion across varying fitness levels (Fletcher et al., 2013; Stevenson et al., 2024; Williams‐Bell et al., 2010). Each sprint began at a fixed speed of 12 km/h and an initial incline of 10%. The incline increased stepwise by two percentage points every 30 s, progressing through 12%, 14% and ultimately 16%, at which point the grade remained constant until exhaustion. Total work performed during exercise was quantified as the cumulative elevation gain across the three sprints because it integrates exercise duration and achieved incline. This provides a sensitive measure of performance under stepwise increasing mechanical load. Thereby it allows direct comparison across repeated sprints with identical speed and incline profiles whilst allowing an increasing load profile to maintain a short but not negligible exercise duration for a wide variety of subjects across different fitness levels.

**FIGURE 1:**
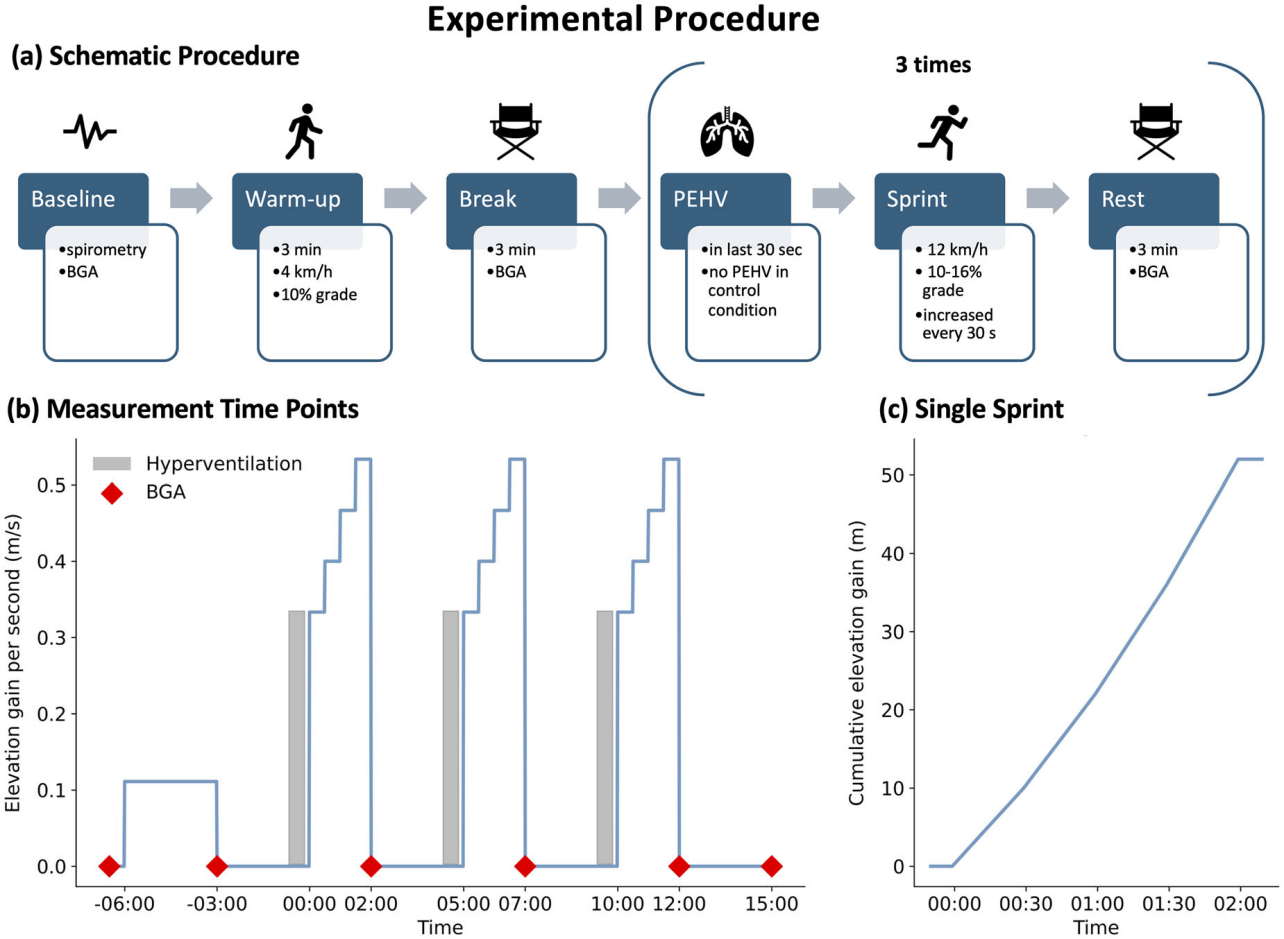
Experimental procedure. (a) Schematic illustration of the procedure. (b) Measurement time points. Participants first underwent a baseline measurement for blood gas analysis (BGA) and spirometry. A 3‐min warm‐up at 4 km/h and 10% grade followed. Afterward, participants had a 3‐min break during which a BGA measurement was taken. For the pre‐exercise hyperventilation (PEHV) condition, participants performed hyperventilation for the last 30 s of rest. In the control condition, the break lasted 3 min without hyperventilation. The sprint phase then began at 12 km/h with an initial 10% incline, increasing automatically by 2% every 30 s until reaching 16% after 1:30 min. The incline then remained constant. (c) The elevation gain during a single sprint. Immediately after each sprint, a BGA was conducted. Following the third sprint participants remained seated for an additional 3 min, after which a final BGA was conducted.

Termination occurred either upon the participant's signal or at the discretion of the study team based on safety criteria (participants nearing the end of the treadmill belt, gripping the handrails or exhibiting visible signs of complete exhaustion). In the pre‐exercise hyperventilation condition, participants performed controlled hyperventilation during the last 30 s before each sprint. BGA was conducted after each sprint. After completion of the third sprint, participants remained seated for an additional 3 min, after which a final BGA was performed (Figure 1).

#### 2.3.1 Design of the breathing protocol

**FIGURE 2:**
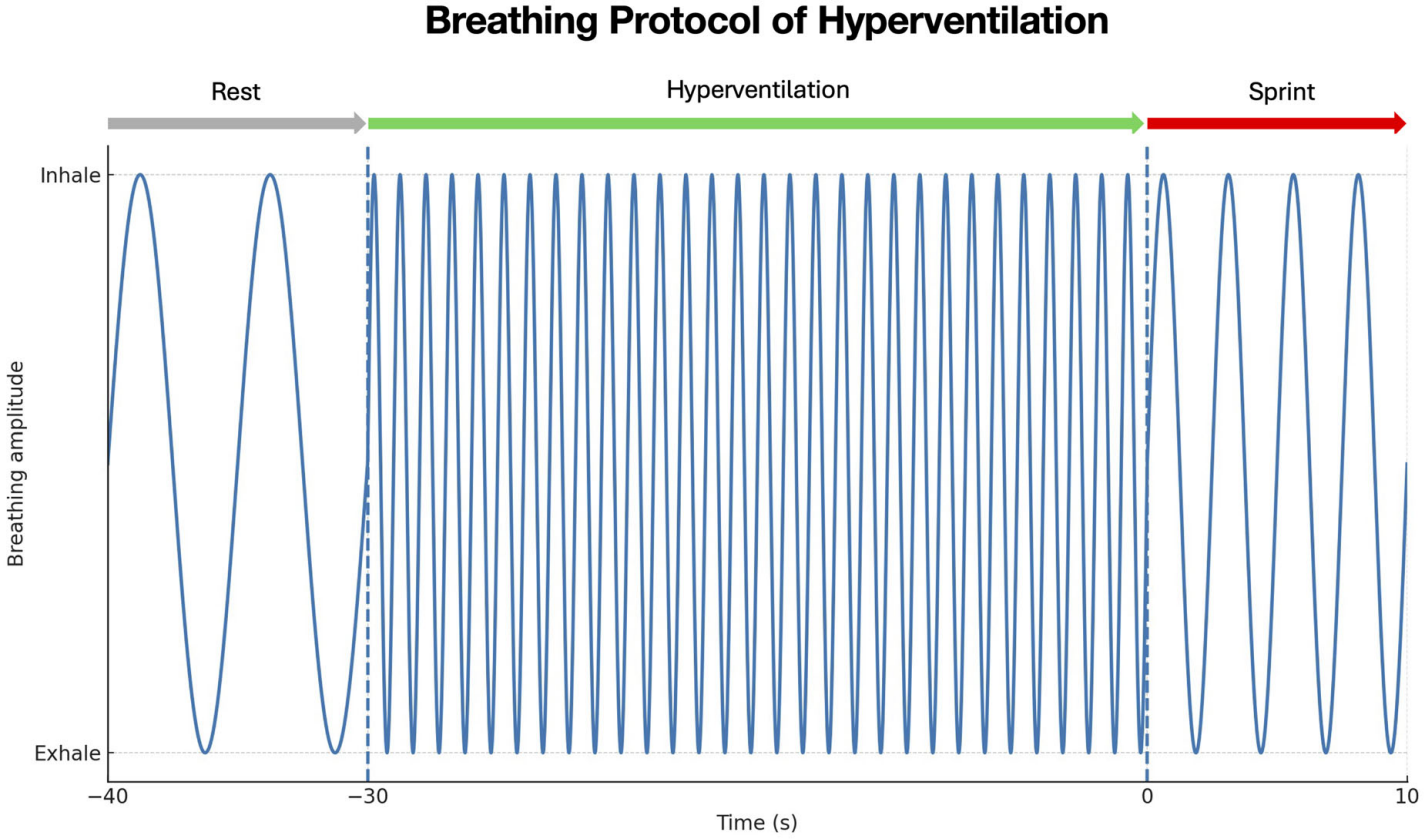
Breathing protocol of hyperventilation. Participants performed 30 s of controlled hyperventilation at 60 breaths/min, immediately followed by the onset of the sprint.

We selected the pre‐exercise hyperventilation protocol described by Sakamoto et al. because it is easily implementable in real‐world settings and has been shown to consistently and meaningfully reduce end‐tidal CO_2_ partial pressure ($P_{\mathrm{ETC}O_{2}}$) (Sakamoto et al., 2014, 2018). This pre‐exercise hyperventilation breathing protocol consisted of 30 s of controlled hyperventilation at a breathing rate of 60 breaths/min. A metronome at 120 bpm cued a command to inhale and exhale alternately, resulting in the desired breathing rate. To ensure adherence to the breathing frequency and technique, participants practiced the hyperventilation beforehand under supervision. We instructed the participants to maintain continuous, deep abdominal breathing. A study team member synchronized their breathing with the participants to support proper execution. Breathing frequency and exhaled CO_2_ levels were continuously monitored via spirometry and objectively analysed in real‐time (Figure 2).

### 2.4 Subjective assessment

A subjective assessment was conducted after the second session using a six‐item questionnaire, which was specifically developed for this study. The items assessed overall subjective evaluation of the breathing intervention, ease of implementation, willingness to use the technique before future short sprints, perceived exertion compared to no intervention, perceived performance benefit, and likelihood of recommending pre‐exercise hyperventilation to other athletes. The Likert scale rating ranged from –5 (‘not at all’/‘very difficult’/‘clearly worse’) to +5 (‘very much’/‘very easy’/‘clearly better’).

### 2.5 Data processing and statistical analysis

All analyses were conducted using Python 3.9 and SciPy statistics library (Virtanen et al., 2020). To compare the pre‐exercise hyperventilation and control conditions across repeated measures, we fitted a linear mixed‐effects model including condition, sprint and their interaction as fixed effects, with subject specified as a random effect. This approach accounts for within‐subject correlation and allows assessment of main effects and condition × sprint interactions within a unified analytical framework. Data are presented as means ± standard deviation (SD) for continuous variables, whereas ordinal data are reported as median and interquartile range (IQR).

Maximal oxygen uptake (${\dot{V}}_{O_{2}\max}$) was determined using breath‐by breath spirometry. ${\dot{V}}_{O_{2}\max}$ was assessed as the highest 30 s rolling average of oxygen consumption achieved during the protocol, accompanied by a respiratory exchange rate ≥1.10 and subjective exhaustion (Borg scale ≥17).

The further analysed parameters included elevation gain during the sprints, CO_2_ elimination, $P_{\mathrm{ETC}O_{2}}$, blood lactate, blood pH, bicarbonate and base excess. $P_{\mathrm{ETC}O_{2}}$ was assessed as mean value over a 5‐s period from −10 to −5 s immediately before the start of each sprint bout. In addition, correlations between changes in CO_2_ elimination and changes in elevation gain as well as between changes in $P_{\mathrm{ETC}O_{2}}$ and changes in elevation gain were assessed using Pearson's correlation coefficient.

We considered a two‐tailed *P*‐value <0.05 as statistically significant.

## 3 RESULTS

A total of 36 subjects participated in this study (Table 1).

### 3.1 Spirometry during pre‐exercise hyperventilation

**FIGURE 3:**
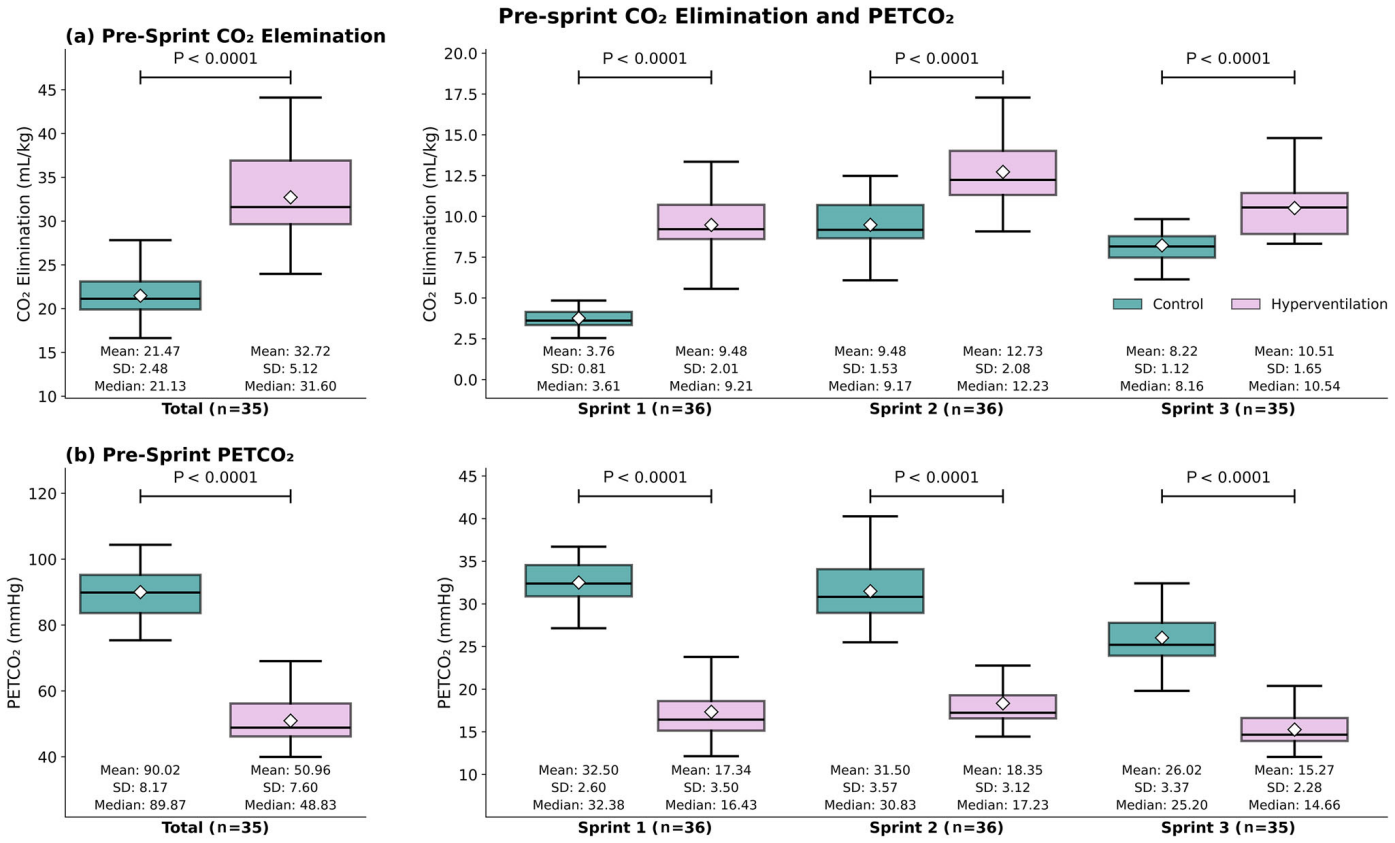
Pre‐sprint CO_2_ elimination and $P_{\mathrm{ETC}O_{2}}$. (a) Total cumulative CO_2_ elimination during the 30 s immediately preceding a sprint across all three sprints (*n* = 35), as well as for each single sprint bout (sprint 1 and sprint 2: *n* = 36; sprint 3: *n* = 35) for both the control (green) and the pre‐exercise hyperventilation condition (pink). (b) $P_{\mathrm{ETC}O_{2}}$ during the 30 s immediately preceding sprint 1, 2 and 3 as well as across all three sprints for both the control (green) and the pre‐exercise hyperventilation (pink) condition (values of *n* same as in (a)). In the pre‐exercise hyperventilation condition, hyperventilation was performed for the whole 30 s immediately preceding the sprints.

Mean CO_2_ elimination was increased during the 30 s of hyperventilation compared to control cumulative value across all sprints (32.72 ± 5.12 mL/kg vs. 21.47 ± 2.48 mL/kg, mean difference (MD) = 11.25 [95% confidence interval (CI): 10.03, 12.29], *P* < 0.0001, *n* = 35), prior to the first sprint (9.48 ± 2.01 mL/kg vs. 3.76 ± 0.81 mL/kg, MD = 5.714 [95% CI: 5.11, 6.31], *P* < 0.0001, *n* = 36), prior to the second sprint (12.73 ± 2.08 mL/kg vs. 9.48 ± 1.53 mL/kg, MD = 3.25 [95% CI: 2.65, 3.85], *P* < 0.0001, *n* = 36), as well as prior to the third sprint (10.51 ± 1.65 mL/kg vs. 8.22 ± 1.12 mL/kg, MD = 2.29 [95% CI: 1.69, 2.89], *P* < 0.0001, *n* = 35) (Figure 3).

The corresponding $P_{\mathrm{ETC}O_{2}}$ at the end of hyperventilation was reduced compared to control cumulative value across all three sprints (50.96 ± 7.60 mmHg vs. 90.02 ± 8.17 mmHg, MD = −39.06 [95% CI: −40.69, −37.16], *P* < 0.0001, values of *n* are the same as for mean CO_2_ elimination), prior to the first sprint (17.34 ± 3.50 mmHg vs. 32.50 ± 2.60 mmHg, MD = −15.16 [95% CI: −16.26, −14.06], *P* < 0.0001), prior to the second sprint (18.35 ± 3.12 mmHg vs. 31.50 ± 3.57 mmHg, MD = −13.15 [95% CI: −14.25, −12.05], *P* < 0.0001), and prior to the third sprint (15.27 ± 2.28 mmHg vs. 26.02 ± 3.37 mmHg, MD = −10.75 [95% CI: −11.85, −9.66], *P* < 0.0001) (Figure 3).

### 3.2 Sprint performance (*n* = 36)

**FIGURE 4:**
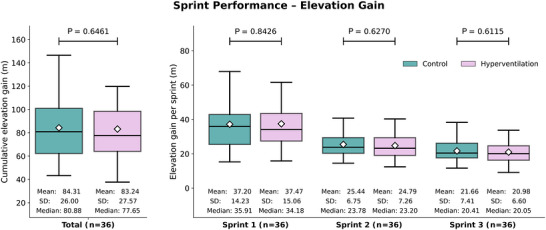
Sprint performance – elevation gain. The figure shows elevation gain in metres for the control (green) and pre‐exercise hyperventilation (pink) condition cumulative across all three sprints, for sprint 1, sprint 2 and sprint 3 (*n* = 36).

Mean cumulative elevation gain across all three sprints did not differ between pre‐exercise hyperventilation (83.24 ± 27.57 m) and control condition (84.31 ± 26.00 m, MD = −1.07 [95% CI: −5.62, 3.49], *P* = 0.6461). Additionally, we observed no difference in mean elevation gain between hyperventilation and control condition for sprint 1 (37.47 ± 15.06 m vs. 37.20 ± 14.23 m, MD = 0.27 [95% CI: −2.36, 2.90], *P* = 0.8426), sprint 2 (24.79 ± 7.26 vs. 25.44 ± 6.75 m, MD = −0.65 [95% CI: −3.28, 1.98], *P* = 0.6270) and sprint 3 (20.98 ± 6.60 m vs. 21.66 ± 7.41 m, MD = −0.68 [95% CI: −3.31, 1.95], *P* = 0.6115) (Figure 4).

### 3.3 Relation of pre‐exercise hyperventilation and performance

**FIGURE 5:**
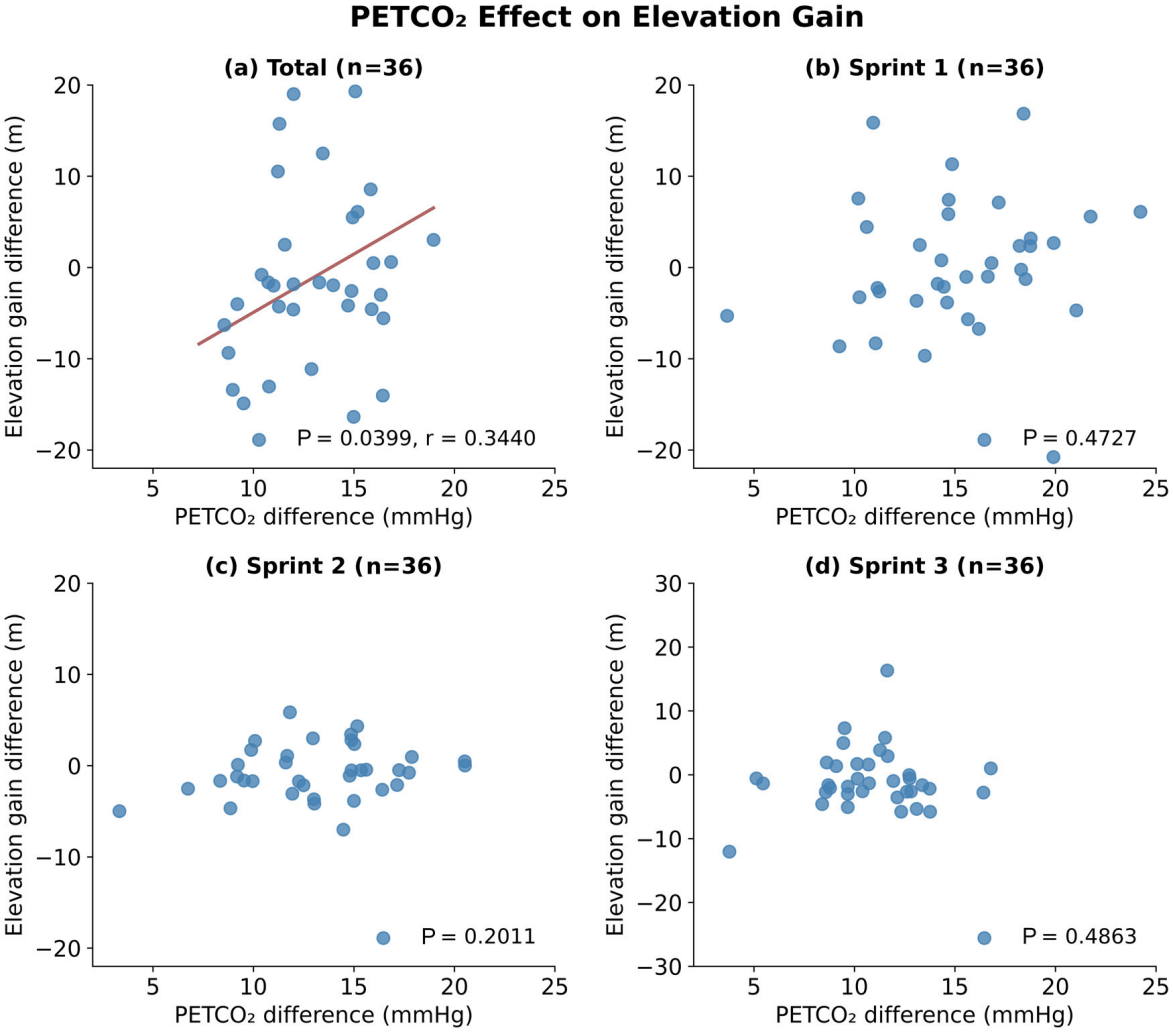
$P_{\mathrm{ETC}O_{2}}$ effect on elevation gain. The figure depicts the relationship between difference in $P_{\mathrm{ETC}O_{2}}$ (hyperventilation minus control) and difference in elevation gain (hyperventilation minus control) cumulative across all three sprints (a; *n* = 35, for sprint 1 (b; *n* = 36), sprint 2 (c; *n* = 36) and sprint 3 (d; *n* = 35).

We found a positive correlation for $P_{\mathrm{ETC}O_{2}}$ difference (hyperventilation minus control) and difference in elevation gain (hyperventilation minus control) cumulative value across all sprints (*r* = 0.3440, *P* = 0.0399, *n* = 35). There was no observable, sprint‐specific correlation between difference in $P_{\mathrm{ETC}O_{2}}$ and difference in elevation gain for the first (*P* = 0.4727, *n* = 36), second (*P* = 0.2011, *n* = 36), or third sprint (*P* = 0.4863, *n* = 35) (Figure 5).

We observed no correlation between the difference in CO_2_ elimination (hyperventilation minus control) and the corresponding elevation gain difference (hyperventilation minus control) for sprint 1 (*P* = 0.1823, *n* = 36), sprint 2 (*P* = 0.2431, *n* = 36), sprint 3 (*P* = 0.9869, *n* = 36) or the cumulative value across all sprints (*P* = 0.5905, *n* = 35).

### 3.4 Metabolic responses

**FIGURE 6:**
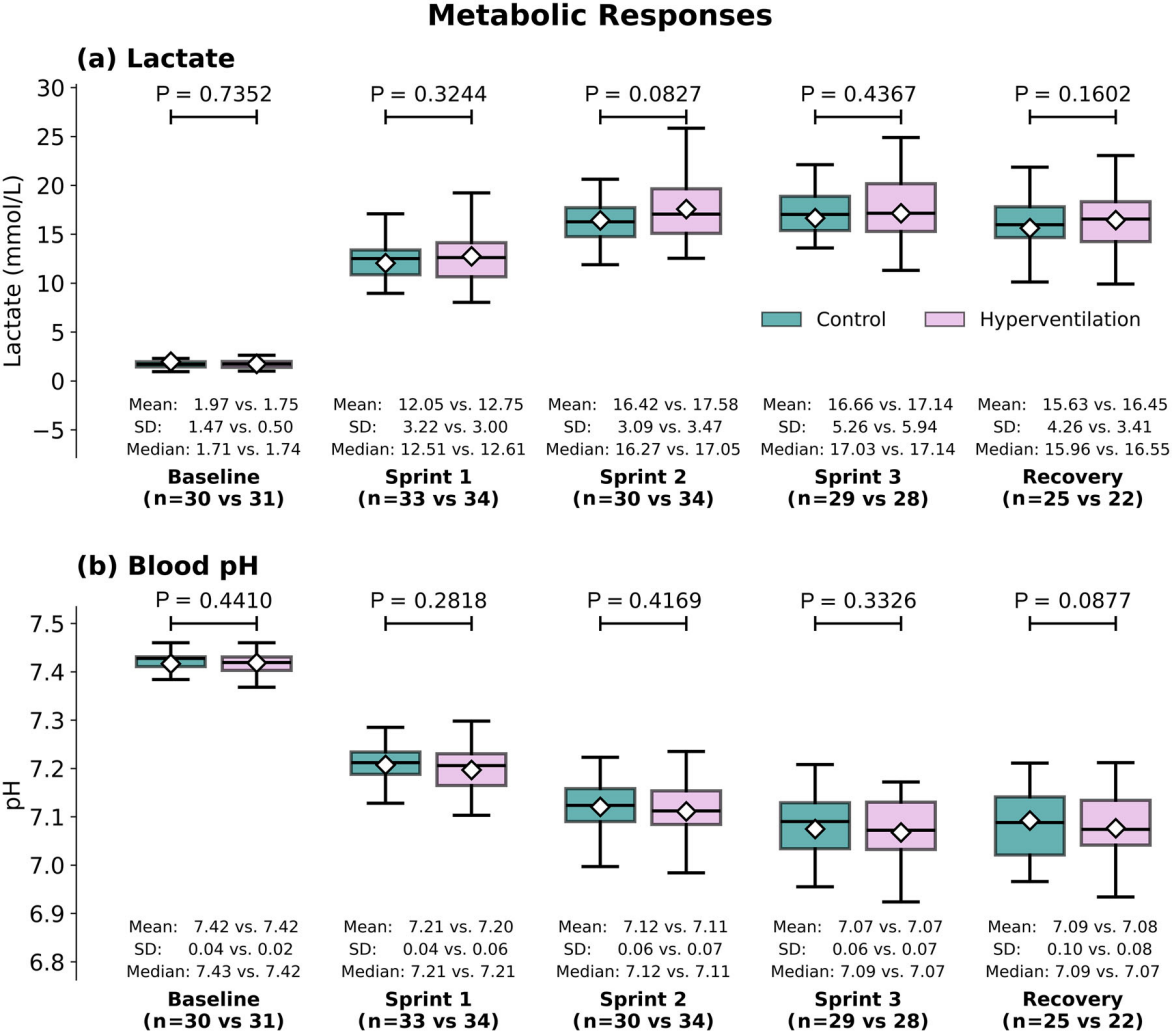
Metabolic responses. The figure shows blood lactate (a, upper panel) and pH (b, lower panel) level at baseline (*n* = 30 vs. 31), after sprint 1 (*n* = 33 vs. 34), sprint 2 (*n* = 30 vs. 34), sprint 3 (*n* = 29 vs. 28), and recovery (3 min after end of sprint 3; *n* = 25 vs. 22). For each time point, values are presented separately for the control (green) and the pre‐exercise hyperventilation (pink) condition.

**FIGURE 7:**
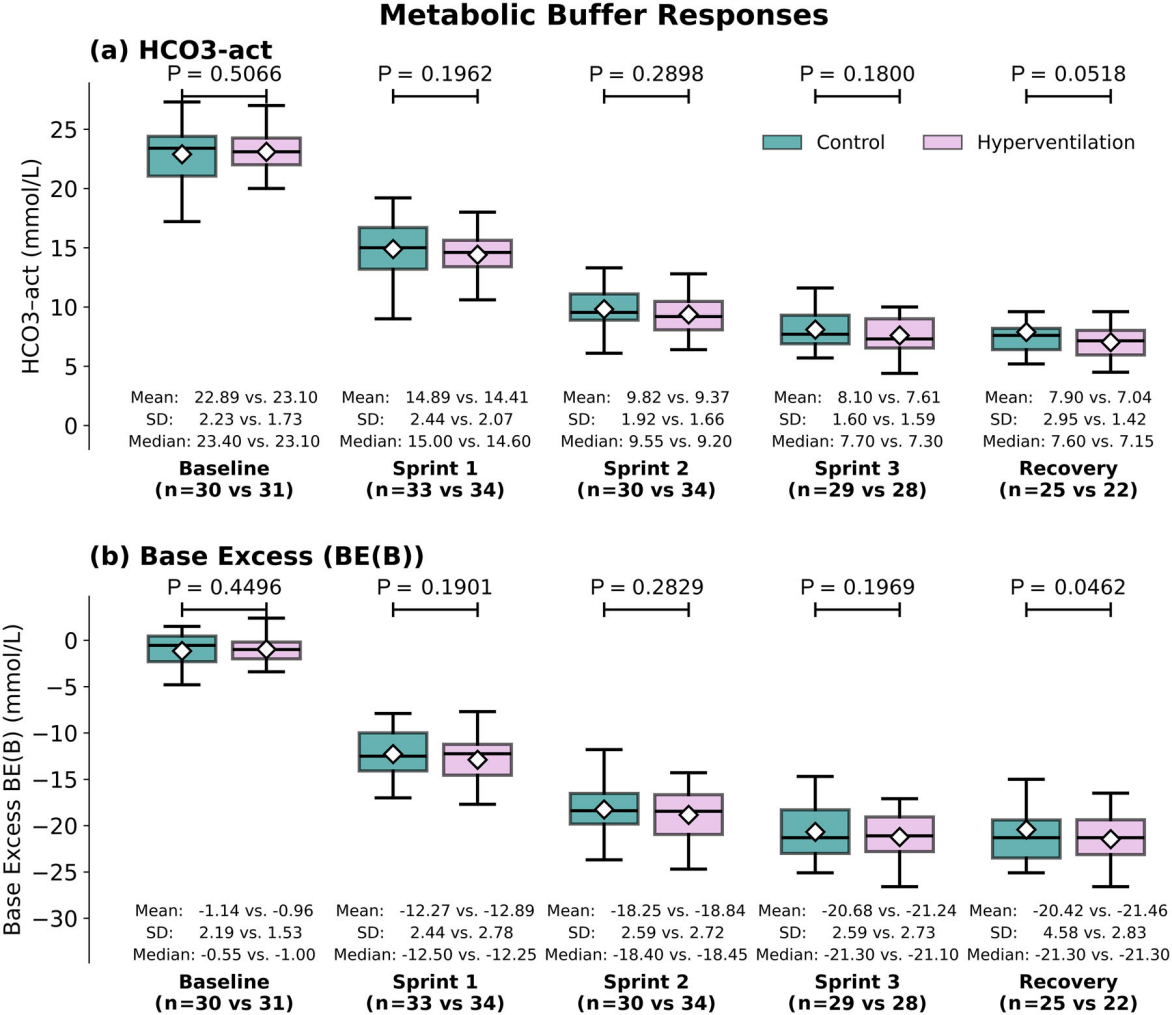
Metabolic buffer responses. The figure shows bicarbonate (a, upper panel) and base excess (b, lower panel) level at baseline (*n* = 30 vs. 31), after sprint 1 (*n* = 33 vs. 34), sprint 2 (*n* = 30 vs. 34), sprint 3 (*n* = 29 vs. 28), and recovery (3 min after end of sprint 3; *n* = 25 vs. 22). For each time point, values are presented separately for the control (green) and the pre‐exercise hyperventilation (pink) condition.

For mean blood lactate (mmol/L) we found no changes between pre‐exercise hyperventilation and control condition for baseline (1.75 ± 0.50 vs. 1.97 ± 1.47, *P* = 0.7352; *n* = 30 vs. 31), sprint 1 (12.75 ± 3.00 m vs. 12.05 ± 3.22 m, *P* = 0.3244 *n* = 33 vs. 34), sprint 2 (17.58 ± 3.47 m vs. 16.42 ± 3.09, *P* = 0.0828 *n* = 30 vs. 34), sprint 3 (17.14 ± 5.94 vs. 16.66 ± 5.26, *P* = 0.4367; *n* = 29 vs. 28) and recovery (16.45 ± 3.41 vs. 15.63 ± 4.26, *P* = 0.1602; *n* = 25 vs. 22) (Figure 6, Figure 7).

Similarly, no alterations in mean blood pH were observed between pre‐exercise hyperventilation and control condition for baseline (7.42 ± 0.02 vs. 7.42 ± 0.04, *P* = 0.4410; values of *n* are the same as for blood lactate), sprint 1 (7.20 ± 0.06 m vs. 7.21 ± 0.04, *P* = 0.2818), sprint 2 (7.11 ± 0.07 m vs. 7.12 ± 0.06 m, *P* = 0.4169), sprint 3 (7.07 ± 0.07 vs. 7.07 ± 0.06, *P* = 0.3326) and recovery (7.08 ± 0.08 m vs. 7.09 ± 0.10, *P* = 0.0877) (Figure 6).

### 3.5 Metabolic buffer responses

For bicarbonate (HCO_3_^−^, mmol/L) we observed no differences between the pre‐exercise hyperventilation and control conditions for baseline (23.10 ± 1.73 vs. 22.89 ± 2.23, MD = 0.28, 95% CI [−0.54, 1.09], *P* = 0.5066, values of *n* are the same as for blood lactate), sprint 1 (14.41 ± 2.07 vs. 14.89 ± 2.44, MD = −0.51 [95% CI: −1.28, 0.26], *P* = 0.1962), sprint 2 (9.37 ± 1.66 vs. 9.82 ± 1.92, MD = −0.43 [95% CI: −1.22, 0.36], *P* = 0.2898), sprint 3 (7.61 ± 1.59 vs. 8.10 ± 1.60, MD = −0.58 [95% CI: −1.42, 0.266], *P* = 0.1800) and recovery (7.04 ± 1.42 vs. 7.90 ± 2.95, MD = −0.92 [95% CI: −1.86, 0.01], *P* = 0.0518).

For base excess (mmol/L), values were lower in the pre‐exercise hyperventilation condition than in the control condition during recovery (−21.46 ± 2.83 vs. −20.42 ± 4.58, MD = −1.21 [95% CI: −2.39, −0.02], *P* = 0.0462, values of *n* are the same as for blood lactate). No differences were observed at baseline (−0.96 ± 1.53 vs. −1.14 ± 2.19, MD = 0.40 [95% CI: −0.64, 1.43], *P* = 0.4496), sprint 1 (−12.89 ± 2.78 vs. −12.27 ± 2.44, MD = −0.66 [95% CI: −1.63, 0.33], *P* = 0.1901), sprint 2 (−18.84 ± 2.72 vs. −18.25 ± 2.59, MD = −0.55 [95% CI: −1.56, 0.46], *P* = 0.2819), and sprint 3 (−21.24 ± 2.73 vs. −20.68 ± 2.59, MD = −0.71 [95% CI: −1.78, 0.37], *P* = 0.1969) (Figure 7).

### 3.6 Subjective assessment

**FIGURE 8:**
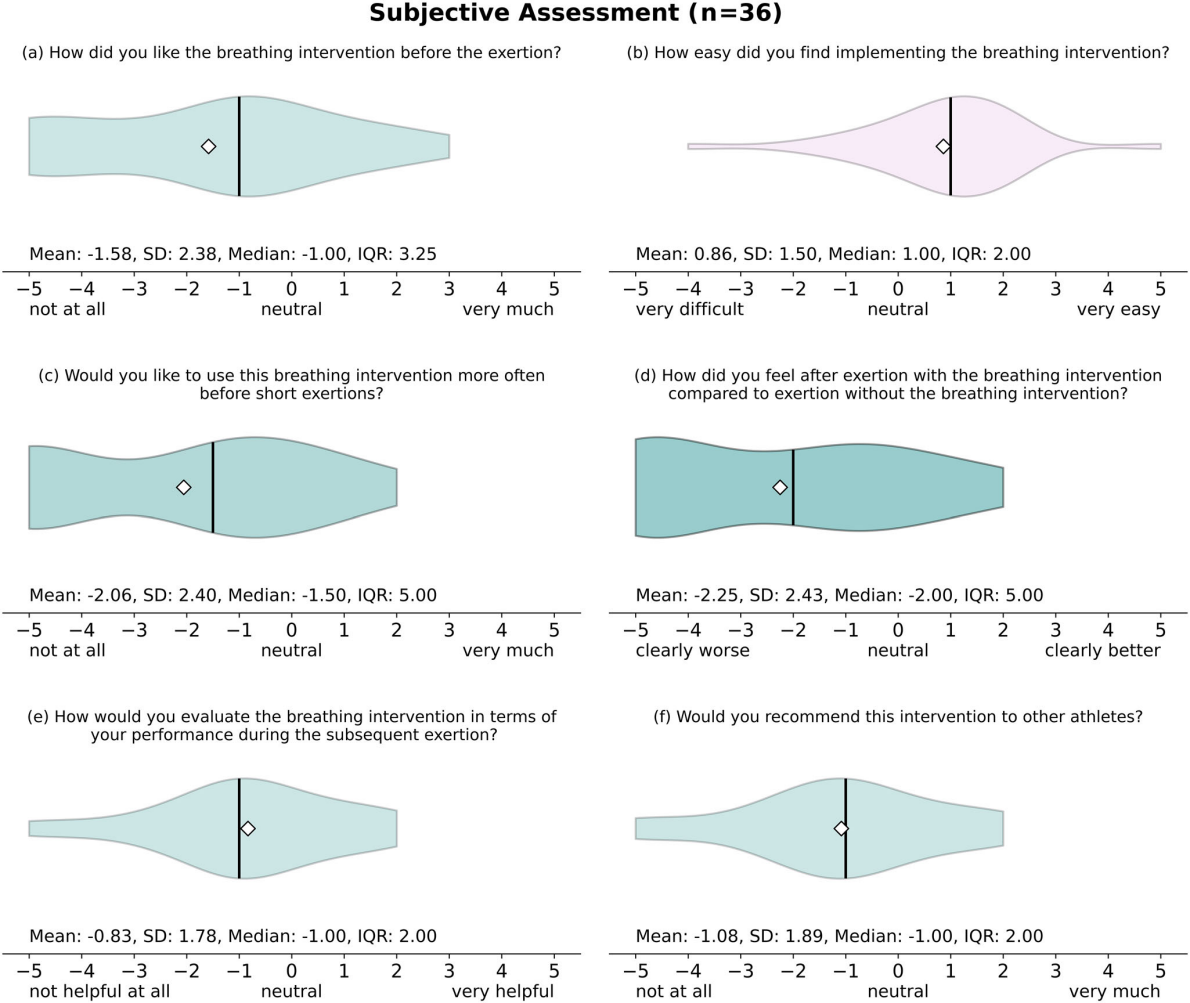
Subjective assessment. The figure shows violin plots of all 36 participants’ ratings of the pre‐exercise hyperventilation across six different domains after completing both sessions: (a) overall liking of the breathing intervention, (b) ease of implementation, (c) willingness to use before future short sprints, (d) perceived exertion compared with no intervention, (e) perceived performance benefit, (f) likelihood of recommending the technique to other athletes. Each plot shows the full distribution in a violin plot, median (vertical line) and mean (diamond); ratings range from –5 (‘not at all’/‘very difficult’/‘clearly worse’) to +5 (‘very much’/‘very easy’/‘clearly better’). IQR, interquartile range.

Thirty‐six subjects completed the subjective assessment questionnaire. While finding it easy to implement (1.00, 2.00, scale −5 to +5), most participants did not like the pre‐exercise hyperventilation breathing protocol (−1.00, 3.25). Subjects would not like to use it more often before short exertions (−1.50, 5.00) and felt worse afterwards compared to the exertion under control condition (−2.00, 5.00). Furthermore, participants did not think that the breathing intervention was helpful in terms of their performance (−0.83, 2.00) and would not recommend it to other athletes (−1.00, 2.00) (Figure 8).

## 4 DISCUSSION

In this randomized, controlled crossover study, we investigated the effect of real‐world applicable, short‐term, pre‐exercise hyperventilation on repeated incline sprint performance in healthy recreational athletes.

Pre‐exercise hyperventilation resulted in increased CO_2_ elimination and lower $P_{\mathrm{ETC}O_{2}}$, but did not alter sprint performance. Comparing pre‐exercise hyperventilation with the control condition, we found no correlation between the difference in CO_2_ elimination and the difference in elevation gain. Only differences in $P_{\mathrm{ETC}O_{2}}$ and elevation gain across all three sprints showed a medium‐strength correlation. We observed no difference in blood lactate, bicarbonate and pH comparing both conditions. We observed a lower base excess in the hyperventilation condition during recovery compared to the control condition. Subjective assessment via questionnaire revealed that participants found the hyperventilation protocol unpleasant, felt worse afterwards and would not recommend its use.

### 4.1 Physiological mechanisms

The marked increase in CO_2_ elimination and reduction in $P_{\mathrm{ETC}O_{2}}$ in pre‐exercise hyperventilation confirmed successful hyperventilation. Moreover, $P_{\mathrm{ETC}O_{2}}$ values were lower in this study than those reported in comparable protocols, which indicates successful achievement of relevant hyperventilation (Dobashi et al., 2021; Sakamoto et al., 2014). No BGA was performed immediately after the hyperventilation phase due to practical reasons, which may have captured a transient alkalosis. However, pre‐exercise hyperventilation did not lead to an alteration in sprint performance compared to control condition.

Although acidosis itself represents a limiting factor to performance, other mechanisms also become relevant during repeated high‐intensity efforts (Cairns & Lindinger, 2025; Collins et al., 2018). These include neuromuscular fatigue, depletion of phosphocreatine stores, impaired excitation–contraction coupling, and central nervous system fatigue (Bishop, 2012; Bogdanis et al., 1996; Girard et al., 2011)

To ensure real‐world applicability, we kept the pre‐exercise hyperventilation simple and short (Dobashi et al., 2021; Sakamoto et al., 2014). We did observe a correlation between reduction in $P_{\mathrm{ETC}O_{2}}$ and the increase in cumulative elevation gain across all sprints. This finding suggests that some participants who achieved greater hypocapnia tended to perform slightly better overall. However, as this modest correlation was only evident in cumulative data and absent in separate analyses of single sprints, it remains unclear whether the observed correlation represents a true, causal effect or whether it is a masked artefact of additional performance factors such as possible placebo or daily performance variation. This analysis was applied to distinguish performance improvement from decline without implying physiological or clinical relevance, as small differences in cumulative elevation gain should not be interpreted as meaningful effects.

There was no difference in post‐sprint blood lactate or pH between the two conditions, either when analysed cumulatively across all sprints or for each sprint separately. Although, base excess was lower during recovery in the hyperventilation condition, indicating a greater base deficit, post‐sprint pH and lactate were unchanged. This is likely due to concurrent changes in respiratory and metabolic components of acid–base balance (Langer et al., 2022). The intermittent capillary blood sampling protocol may have missed transient alkalosis immediately after hyperventilation.

Hypocapnia induced by pre‐exercise hyperventilation may shift the oxygen–haemoglobin dissociation curve to the left (the Bohr effect). This leftward shift could impair oxygen unloading to the muscle, potentially offsetting any theoretical performance benefits of pre‐exercise hyperventilation (Benner et al., 2025). Furthermore, hypocapnia is associated with cerebral vasoconstriction, which may reduce central motor drive, thereby negatively influencing perceived exertion and performance (Floyd et al., 2003; Yamaguchi et al., 1979). In addition, the extracellular buffering capacity is large compared with the small alkalosis that can be achieved with short‐duration hyperventilation (Weston et al., 1996). As a result, any additional buffering reserve is likely to be rapidly overwhelmed by the acid load generated at the onset of exercise, particularly during repeated high‐intensity efforts.

Participants rated pre‐exercise hyperventilation as neither beneficial nor useful and would not recommend it to others. The fundamentally negative perception of participants towards this simple and brief hyperventilation protocol highlights practical challenges for real‐world application of such breathing techniques even if they were found to improve performance.

### 4.2 Comparison with previous literature

Current scientific literature shows inconsistent results regarding the efficacy of pre‐exercise hyperventilation on high‐intensity loads. Several studies have applied longer pre‐exercise hyperventilation protocols. While some breathing protocols induced a more pronounced respiratory alkalosis, their effects on performance remain inconsistent. The available evidence does not suggest a clear dose–response relationship between the duration or depth of pre‐exercise hyperventilation and subsequent performance outcomes. Instead, factors such as exercise modality, exercise duration and recovery structure appear to be important (Fujii et al., 2015; Johnson et al., 2021; Leithäuser et al., 2016; Morrow et al., 1988; Sakamoto et al., 2014, 2018). Within this context, the present study findings are consistent with the broader literature and are unlikely to be explained solely by the comparatively short and pragmatically designed hyperventilation protocol.

Exercise protocols in studies reporting a performance benefit of pre‐exercise hyperventilation typically involved short sprints with longer recovery periods in between. Previous studies suffered from methodological limitations such as limited generalizability, small sample sizes, lack of blinding, critical susceptibility to placebo effects, and homogeneous participant groups (Fujii et al., 2015; Johnson et al., 2021; Leithäuser et al., 2016; Morrow et al., 1988; Sakamoto et al., 2014, 2018). Furthermore, they were conducted under highly sterilized, laboratory conditions with limited real‐world applicability. Lastly and most importantly, the performance gain was at best modest, raising questions about their practical relevance in real‐world scenarios (Dobashi et al., 2021; Leithäuser et al., 2016; Sakamoto et al., 2014). Studies showing no benefit of pre‐exercise hyperventilation applied shorter hyperventilation durations, as well as exercise protocols with brief recovery periods and high mechanical demand. While showing greater real‐world applicability, the breathing protocols applied in these studies may result in substantially smaller effects, if any at all (Stevenson et al., 2024; Williams‐Bell et al., 2010)

Across studies reporting no performance benefit of pre‐exercise hyperventilation, reductions in $P_{\mathrm{ETC}O_{2}}$ and increases in pH level were mostly transient and rapidly attenuated once high‐intensity exercise commenced (Leithäuser et al., 2016; Stevenson et al., 2024; Williams‐Bell et al., 2010). In studies employing short hyperventilation durations and brief recovery intervals, $P_{\mathrm{ETC}O_{2}}$ has been shown to normalize within the first exercise bout, while blood pH declines likely due to high rates of glycolytic flux and CO_2_ production (Sakamoto et al., 2014, 2018). Any possible alkalinizing effect induced prior to exercise is likely overridden by exercise‐induced metabolic acidosis early during the protocol. Therefore, shorter and more pragmatic breathing interventions appear insufficient to sustain meaningful alterations in acid–base balance throughout repeated high‐intensity efforts (Cairns & Lindinger, 2025; Dobashi et al., 2021; Leithäuser et al., 2016; Stevenson et al., 2024; Williams‐Bell et al., 2010).

### 4.3 Limitations

Despite enrolling a larger cohort than previous investigations, the present study remains limited by a small number of participants, which may have limited statistical power to detect possible subtle performance effects. Blinding was not feasible, allowing potential placebo or motivational effects to influence outcomes. Capillary BGA was intermittent, and pH immediately after pre‐exercise hyperventilation was not measured. This approach was chosen for practical reasons to avoid any delay between the end of the pre‐exercise hyperventilation and the onset of the sprints that could have interfered with potential performance effects. Although the reduction in $P_{\mathrm{ETC}O_{2}}$ following hyperventilation clearly indicates the presence of hypocapnia, the magnitude of the pre‐exercise hyperventilation‐induced change in blood pH cannot be determined within the present study design. This represents a relevant methodological limitation of the study and should be considered when interpreting the acid–base findings.

Future studies could focus on different, target‐controlled breathing protocols while maintaining real‐world applicability. Further, they should evaluate previously reported effects in larger, more heterogeneous groups to improve external validity and comprehensively characterize underlying acid–base kinetics. To minimize potential unblinding effects, future studies could include a sham breathing exercise in the control condition.

### 4.4 Future directions

Potential future applications of pre‐exercise hyperventilation primarily lie in the context of short‐duration, high‐intensity exercises, where rapid modulation of acid–base balance could theoretically influence performance. When considering pre‐exercise hyperventilation in swimming, potential performance‐related intentions must be weighed against well‐documented safety risks, including an increased risk of shallow‐water blackout due to hypocapnia‐induced suppression of the ventilatory drive. Therefore, before any application of pre‐exercise hyperventilation can be recommended, real‐world settings further research is required.

### 4.5 Conclusion

In this randomized crossover study, an easily translatable pre‐exercise hyperventilation protocol did not improve performance in repeated high‐intensity incline sprint intervals. Although the breathing technique lowered $P_{\mathrm{ETC}O_{2}}$ and increased CO_2_ elimination, there was no change in post‐sprint pH and lactate compared to control condition. We observed a moderate correlation for the differences in $P_{\mathrm{ETC}O_{2}}$ and cumulative elevation gain across all sprints. Participants rated pre‐exercise hyperventilation as not helpful and would not recommend it to others. Therefore, we do not recommend the use of this pre‐exercise hyperventilation protocol in real‐world scenarios. Future research could focus on different, target‐controlled breathing protocols while maintaining real‐world applicability.

## AUTHOR CONTRIBUTIONS

Research governance for this study is held by Tomas Lucca Bothe, Oliver Opatz and Andreas Patzak. Tomas Lucca Bothe conceived the study and designed the methodology. Leon Fesseler, Viktor Heinz, Ngan‐Ha Stella Le and Tomas Lucca Bothe collected and curated the data. Leon Fesseler and Tomas Lucca Bothe performed the statistical analysis. Leon Fesseler and Niklas Pilz drafted the manuscript. All authors contributed to reviewing and editing the manuscript. All authors have read and approved the final version of this manuscript and agree to be accountable for all aspects of the work in ensuring that questions related to the accuracy or integrity of any part of the work are appropriately investigated and resolved. All persons designated as authors qualify for authorship, and all those who qualify for authorship are listed.

## CONFLICT OF INTEREST

The authors declare no conflicts of interest.

## Supporting information



Supplementary table legends.

Supplementary Tables S1–S5: raw data from the study.

## Data Availability

The datasets generated and analysed during the current study are available from the corresponding author (L.F.) on reasonable request.
