# Supplementary material for: The effect of pre‐exercise hyperventilation on repeated high‐intensity inclined sprint performance
Source: Exp Physiol. 2026 May 25;111(7):3412–24. doi: 10.1113/EP093393 (PMC13327302; doi:10.1113/EP093393)
Supplement: Supplementary file 1 — Supplementary table legends. [file EPH-111-3412-s002.docx]

**Supporting Information for online publication**

**Supplement 1: Blood Gas Analysis (Hyperventilation condition)**

Raw capillary for blood gas analysis (BGA) data for the hyperventilation condition. For each subject and each measurement time point: Baseline 1 (R1), Baseline 2 (R2), Sprint 1 (1), Sprint 2 (2), Sprint 3 (3), Recovery (R3). Results are shown in Figure 6 and 7.

**Supplement 2: Blood Gas Analysis (Control condition)**

Raw capillary for blood gas analysis (BGA) data for the control condition. For each subject and each measurement time point: Baseline 1 (R1), Baseline 2 (R2), Sprint 1 (1), Sprint 2 (2), Sprint 3 (3), Recovery (R3). Results are shown in Figure 6 and 7.

**Supplement 3: Elevation Gain during Sprint bouts**

Raw data for elevation gain in meters during each sprint and the cumulative elevation gain across all three sprint bouts for both conditions (condition (H) and control (N)). Results are shown in Figure 4.

**Supplement 4: Spirometric Data during Hyperventilation (hyperventilation vs control)**

Raw spirometric data for PETCO₂ calculated as the mean over the five-second interval from -10 to -5 seconds before sprint onset during Hyperventilation (H) and Control (N), and cumulative CO_2_ elimination during the 30-second pre sprint period for Hyperventilation (H) and Control (N). Results are shown in Figure 3 and 5.

**Supplement 5: Body Composition, Fitness, and Sex of all Subjects**

Raw data for all participants including height, weight, sex, and VO₂max. Results are shown in Table 1.
